# UK neonatal stoma practice: a population study

**DOI:** 10.1136/archdischild-2024-327020

**Published:** 2024-06-19

**Authors:** Graciaa Singhal, Rema Ramakrishnan, Raph Goldacre, Cheryl Battersby, Nigel J Hall, Chris Gale, Marian Knight, Nick Lansdale

**Affiliations:** 1 Neonatal Medicine, School of Public Health, Imperial College London, London, UK; 2 National Perinatal Epidemiology Unit, Nuffield Department of Population Health, University of Oxford, Oxford, UK; 3 Unit of Health-Care Epidemiology, Department of Public Health, University of Oxford, Oxford, UK; 4 Centre for Paediatrics and Child Health, Imperial College London, UK, Imperial College London, London, UK; 5 University Surgery Unit, Faculty of Medicine, University of Southampton, Southampton, UK; 6 Department of Paediatric and Neonatal Surgery, Royal Manchester Children's Hospital Department of Paediatric and Neonatal Surgery, Manchester, UK; 7 Division of Developmental Biology & Medicine, Faculty of Biology Medicine and Health, The University of Manchester Faculty of Biology Medicine and Health, Manchester, UK

**Keywords:** Intensive Care Units, Neonatal, Neonatology, Gastroenterology

## Abstract

**Objective:**

The optimal time for neonatal stoma closure is unclear and there have been calls for a trial to compare early and late surgery. The feasibility of such a trial will depend on the population of eligible infants and acceptability to families and health professionals. In this study, we aimed to determine current UK practice and characteristics of those undergoing stoma surgery.

**Design:**

A retrospective cohort study of neonates who had undergone stoma surgery (excluding anorectal malformations and Hirschsprung’s disease) using three national databases: the National Neonatal Research Database (NNRD, 2012–2019), British Association of Paediatric Surgeons Congenital Anomalies Surveillance System (BAPS-CASS, 2013–2014) and Hospital Episode Statistics–Admitted Patient Care (HES-APC, 2011–2018).

**Results:**

1830 eligible neonates were identified from NNRD, 163 from BAPS-CASS, 2477 from HES-APC. Median (IQR) duration of stoma in days was 57 (36–80) in NNRD, 63 (41–130) in BAPS-CASS and 78 (55–122) for neonates identified from HES-APC. At the time of closure, there were low rates of invasive ventilation (13%), inotrope use (5%) and recent steroids use (4%). Infants who underwent earlier closure (<9 weeks) were less preterm (median 28 weeks vs 25 weeks), have higher birth weight (median 986 g vs 764 g) and more likely to have stoma complications (29% vs 5%).

**Conclusion:**

There are sufficient UK neonates undergoing stoma formation for a trial. Stoma closure is performed at around 2 months, with clinical stability, gestation, weight and stoma complications appearing to influence timing. The variation in practice we document indicates there is opportunity to optimise practice through a trial.

**WHAT IS ALREADY KNOWN ON THIS TOPIC**

- Optimal timing for neonatal stoma closure is unclear with conflicting evidence from previous studies.

**WHAT THIS STUDY ADDS**

- This study, the largest and most robust analysis to date, confirms variability in stoma closure timing and infant weight at the time of closure, indicating that a clinical trial to help optimise practice is important.
- Factors such as clinical stability, gestation, weight and stoma complications appear to influence this timing.
- There appears to be a sufficiently large population of preterm neonates with necrotising enterocolitis/spontaneous intestinal perforation undergoing stoma formation to make a UK-based trial feasible.

**HOW THIS MIGHT AFFECT RESEARCH, PRACTICE OR POLICY**

- Data presented here around infant characteristics and outcomes will inform the design of a robust and feasible trial.

## Introduction

 Neonates undergoing emergency abdominal surgery frequently require stomas to be formed. While stomas can be lifesaving, they pose challenges such as fluid and electrolyte imbalance, local wound and skin problems, malnutrition and growth failure, particularly in preterm infants.^1^,^3^ Reversing (closing) these stomas with a second operation is therefore an essential part of the infant’s recovery. The optimal time for stoma closure remains uncertain, with conflicting evidence from published studies and reviews.^12 4^,^6^ While a randomised clinical trial would be the most robust way to determine the optimal timing of closure, such a trial is likely to be challenging due to (1) patient factors such as marked heterogeneity of underlying disease and comorbidities; (2) clinician factors such as equipoise and variable willingness to recruit; and (3) parent factors including trial acceptability.

The Timing of Stoma Closure in Neonates (ToSCiN) study aimed to determine the feasibility of a clinical trial comparing ‘early’ and ‘late’ stoma closure in neonates using an applied mixed methods approach. We have previously reported the viewpoints of UK practitioners on stoma closure timing.^7^ In this study, we used three national databases to determine actual clinical practice around formation and management of neonatal stomas in the UK.

### Aims

The specific aims of this workstream of the ToSCiN study were as follows:

1. Describe the number, indication and patient characteristics of neonates who underwent stoma formation.
2. Describe the timing of stoma closure overall and by two diagnostic subgroups (necrotising enterocolitis (NEC)/spontaneous intestinal perforation (SIP) compared with other intestinal malformations).
3. Describe the characteristics of infants at the time of surgery for stoma closure.
4. Describe factors associated with timing of closure before hospital discharge.

## Methods

We undertook a retrospective cohort study using data from three national databases with individual baby-level data. The databases were the National Neonatal Research Database (NNRD, 1 January 2012–31 December 2019), the British Association of Paediatric Surgeons Congenital Anomalies Surveillance System (BAPS-CASS, NEC study 1 March 2013–28 February 2014 and meconium ileus study 1 October 2012–30 September 2014) and Hospital Episode Statistics–Admitted Patient Care (HES-APC, 1 January 2011–1 December 2018).^8 9^ They varied in geographical, temporal and healthcare setting coverage, but provided some overlapping data items that allowed cross-referencing (table 1). While exact temporal overlap for data extraction from NNRD and HES-APC was not possible, there were seven common years and complete overlap with the two BAPS-CASS studies. A pragmatic approach was taken to define a neonatal stoma: for NNRD, this was a stoma formed during admission to a neonatal unit; for BAPS-CASS, this was a stoma formed for the diagnoses of NEC/SIP or meconium ileus (neonatal conditions); and for HES-APC, this was a stoma formed in the first 90 days of life. Full details of the methodology are provided in the online supplemental methods and tables 1 and 2.

**Table 1:** Indications for stoma formation and summary of data source characteristics

Data source	NNRD	HES-APC	BAPS-CASS
Duration (years included)	8 years (2012–2019)	8 years (2011–2018)	NEC: 1 year (2013–2014)Meconium ileus: 2 years (2012–2014)
Coverage	England/Wales	England	UK and Ireland
Setting	All NHS neonatal units	All NHS settings	Neonatal surgical centres
Total infants			
N	512 964	–	256
Mean per annum	56 996	–	256
Total infants with stoma (any indication)			
n	4368	3541	184
Mean per annum	546	443	184
Anorectal malformations and Hirschsprung’s (planned treatment pathways: excluded)			
n	1083	1064	n/a
Mean per annum	135	133	
Other exclusions (no GI diagnosis, discharge/died before closure, etc)			
n	1455	–	–
NEC/SIP			
n	1543	1537	163
Mean per annum	193	192	163
Other intestinal malformations			
n	287	940	21
Mean per annum	36	118	21

BAPS-CASSBritish Association of Paediatric Surgeons Congenital Anomalies Surveillance SystemGIgastrointestinalHES-APCHospital Episode Statistics–Admitted Patient CareNECnecrotising enterocolitisNNRDNational Neonatal Research DatabaseSIPspontaneous intestinal perforation

## Results

### Indications for stoma and total number of neonates

From the NNRD, 512 964 neonates born in England and Wales between 2012 and 2019 received neonatal care, of whom 4368 (0.9%) had a record of having a stoma (table 1). A total of 1830 neonates met the criteria for inclusion in our analysis (online supplemental figure 1A); 1543 neonates had a diagnosis of NEC/SIP and 287 did not (labelled as having ‘other intestinal malformations’). The list of gastrointestinal diagnoses is shown in table 2. From BAPS-CASS, there were 163 and 21 neonates who had stoma formation in the NEC/SIP and meconium ileus studies, respectively (table 1 and online supplemental figure 1B). From HES-APC, there were 3541 neonates who had stoma formation (table 1). About 2477 neonates met the inclusion criteria: 1537 had a diagnosis of NEC/SIP and 940 had other indications for a stoma (online supplemental figure 1C). Indications for stoma formation for the common year (March 2013–March 2014) are provided in online supplemental table 3.

**Table 2:** Gastrointestinal diagnoses for neonates with a stoma (after exclusions) in England and Wales, within the National Neonatal Research Database, 1 January 2012 to 31 December 2019

NNRD data—List of GI diagnosis		Neonates (n)	% of neonates
NEC/SIP		1543	84.3
Other malformations(n=287)	Gastroschisis	66	3.6
	Meconium ileus	53	2.9
	Volvulus	44	2.4
	Duodenal atresia/stenosis	21	1.1
	Ileal/jejunal atresia	63	3.4
	Small intestine atresia/absence/stenosis/obstruction	108	5.9
	Malrotation/intussusception of intestine	14	0.8

GIgastrointestinalNECnecrotising enterocolitisNNRDNational Neonatal Research DatabaseSIPspontaneous intestinal perforation

### Stoma closure timing

**Figure 1:**
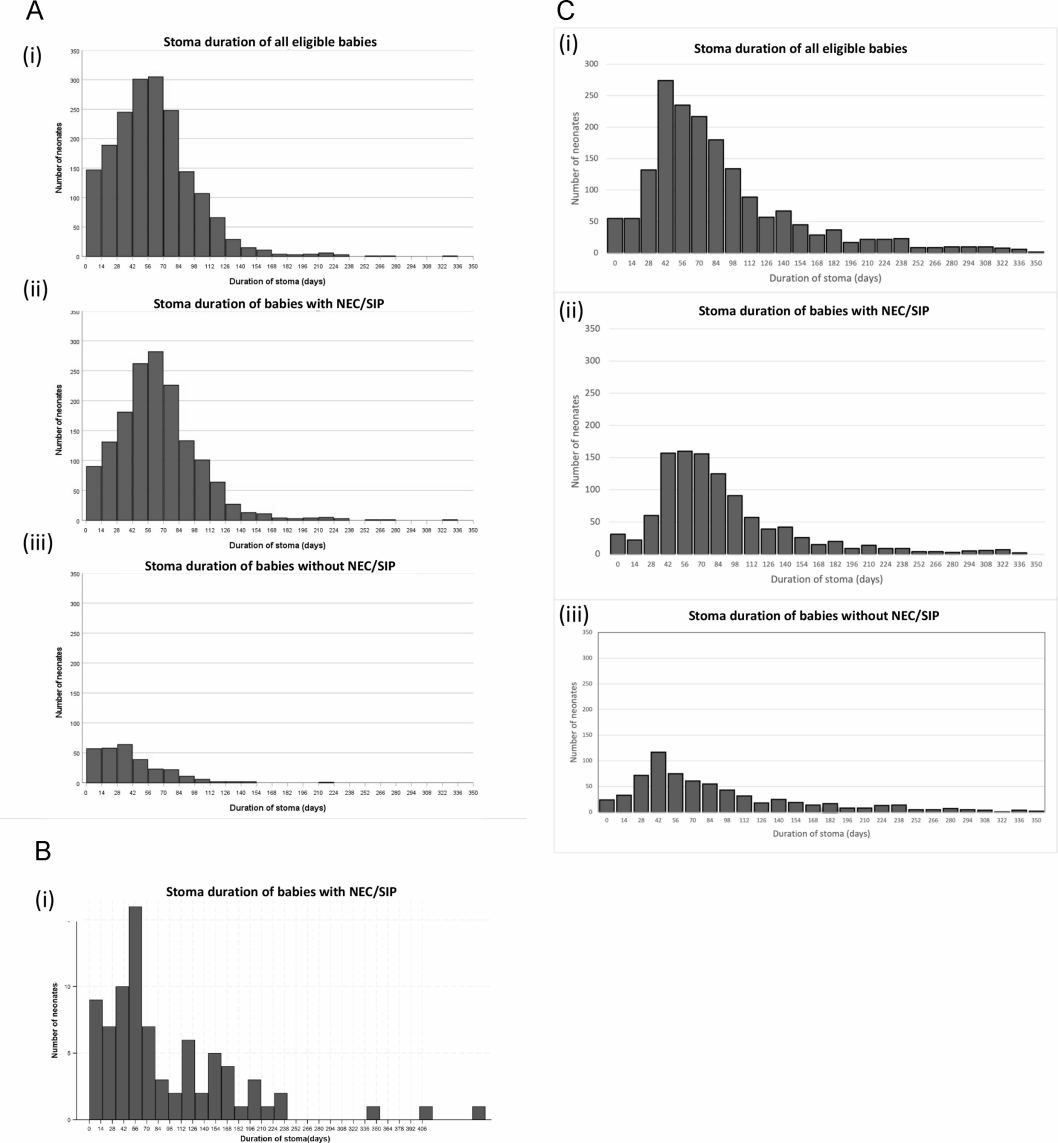
(A) Histograms depicting stoma closure timings for (i) all neonates, (ii) neonates with necrotising enterocolitis/spontaneous intestinal perforation (NEC/SIP) and (iii) other malformations from the National Neonatal Research Database. (B) Histogram depicting stoma closure timing for NEC/SIP infants from British Association of Paediatric Surgeons Congenital Anomalies Surveillance System database. (C) Histograms depicting stoma closure timings for (i) all eligible neonates, (ii) neonates with NEC/SIP and (iii) other malformations from the Hospital Episode Statistics-Admitted Patient Care database.

From the NNRD data, the median (IQR) duration of stoma was 57 (36–80) days for all neonates, 60 (41–83) days for neonates with NEC/SIP and 37 (17–55) days for neonates without NEC/SIP. Histograms depicting the distribution of stoma closure timings are shown in figure 1A.

For BAPS-CASS, 81 infants with NEC/SIP underwent stoma closure, and the median (IQR) duration of stoma was 63 days (41–130) (figure 1B). Twenty infants with meconium ileus underwent stoma closure, with a median (IQR) stoma duration of 51 (36–106) days (online supplemental figure 2).

For HES-APC, the frequency distribution of stoma duration is shown in figure 1C. In patients overall, the median (IQR) number of days to closure was 77 (52 –117); in patients with NEC/SIP, it was 78 (55–122); in patients without NEC/SIP, it was 74 (45–124). When those discharged with a stoma in situ were excluded, the median (IQR) time to closure was 61 days (45–84) overall, 66 days (49–87) in those with NEC/SIP and 51 days (37–73.5) in those without NEC/SIP.

### Neonatal characteristics

#### National Neonatal Research Database

The median birth weight for neonates with NEC/SIP was 860 g, and 2440 g for neonates with other malformations (table 3). Neonates with NEC/SIP were also born more preterm than neonates with other malformations, with median gestational age of 26 and 35 weeks, respectively.

**Table 3:** Characteristics of infants undergoing stoma closure prior to neonatal discharge among infants from the National Neonatal Research Database

	NEC/SIP(n=1543)	Other malformations(n=287)
Birth weight (g) (median, IQR)	860 (695–1190)	2440 (1430–2980)
Gestation age at birth (weeks) (median, IQR)	26 (25–29)	35 (31–37)
Weight at stoma formation (g) (median, IQR)	1150 (820–1657)	2310 (1540–3135)
Age at stoma formation (days) (median, IQR)	18 (9–37)	5 (3–11)
Weight at stoma closure (g) (median, IQR)	2550 (2060–3125)	3150 (2576–3796)
Age at stoma closure (days) (median, IQR)	88 (64–112)	45 (25–72)
Duration of stoma in situ (days) (median, IQR)	60 (41–83)	37 (17–55)
Inotropes 2 days before closure (%, n)	4.5 (69)	2.1 (6)
Parental nutrition 2 days before stoma closure (%, n)	58.1 (897)	65.2 (187)
Ventilation 2 days pre closure (%, n)		
None	45.3 (699)	67.6 (194)
Non-invasive	41.0 (633)	16.0 (46)
Invasive	13.3 (204)	16.4 (47)
Missing	0.6 (10)	0 (0)
Steroids in 7 days before stoma closure (%, n)	3.8 (60)	1.4 (4)

NECnecrotising enterocolitisSIPspontaneous intestinal perforation

The median (IQR) postnatal age (days) at stoma formation for neonates with NEC/SIP was 18 (9–37) and 5 (3–11) for neonates with other malformations; age in days at stoma closure was 88 (64–112) among neonates with NEC/SIP and 45 (25–72) for other malformations, respectively.

Two days prior to stoma closure, among NEC/SIP neonates, 4.5% received inotropes, 58.1% received parenteral nutrition (PN), 41% needed non-invasive ventilation and 13.3% received invasive ventilation (table 3); 3.8% received steroids 7 days before closure. Median (IQR) weight at stoma closure was 2550 g (2060–3125).

A total of 217 neonates were discharged from neonatal care prior to stoma closure, 178 neonates with NEC/SIP and 39 with other malformations (online supplemental table 4); data describing stoma closure were not available for these babies as the NNRD only holds data on neonates during neonatal care and stoma closure occurred outside of neonatal care. In these, neonates the median stoma duration before discharge was 49 days for the NEC/SIP group and 28 days for other malformations.

Furthermore, 209 neonates died prior to stoma closure, 197 neonates with NEC/SIP and 12 with other malformations (online supplemental table 4).

### British Association of Paediatric Surgeons Congenital Anomalies Surveillance System

#### NEC/SIP study

The median (IQR) birth weight for neonates with NEC/SIP was 950 g (745–1375) and gestation (weeks) 27 (25–30). Of the 81 NEC/SIP infants who underwent stoma closure, median (IQR) postnatal age (days) at formation was 17 (8–33) and at closure was 94 (60–154). The median (IQR) length of bowel from the duodeno-jejunal flexure to the stoma was 51 (35–70) cm and stoma complications occurred in 14 (17%). With regard to nutrition after stoma formation, 48 (61%) were still receiving PN at 28 days and 75 (95%) were receiving at least some enteral feeds. A total of 37 (22.7%) infants died during the study period; the majority (n=30) of these occurred within 28 days of initial surgery.

#### Meconium ileus study

Of the 21 infants who underwent stoma closure, the median (IQR) postnatal age (days) at closure was 51 (36–106). For nine infants, this was before discharge from the hospital. The median (IQR) length of stay was 46.5 (23–71.5) days. No infant died during the study period. Nine infants had stoma-related complications within 28 days of surgery and an additional four infants had complications by 1 year post-surgery.

### Hospital Episode Statistics–Admitted Patient Care

The median birth weight for neonates with NEC/SIP was 889 g (705–1400) and 2550 g (1561–3090) for neonates with other malformations. Neonates with NEC/SIP were also born more preterm than neonates with other malformations, with median gestational age of 27 weeks (25–30) and 36 (31–38), respectively.

The median (IQR) postnatal age (days) at stoma formation for neonates with NEC/SIP was 12 (6–31) and 5 (1–28) for neonates with other malformations. Among neonates with NEC/SIP, median age in days at stoma closure was 100 (76–134) or 88 (68–110) when excluding patients discharged with stoma in situ. Among neonates with other malformations, median age at stoma closure was 90 (53–148) or 62 (42–93) days when excluding patients discharged with stoma in situ. In total, 466 (18.8%) patients died in infancy; the majority (n=404) of these occurred before stoma closure. Among the subgroup of patients with NEC/SIP, 355 (23.1%) died in infancy (307 before stoma closure). Among the subgroup of patients without NEC/SIP, 111 (11.8%) died in infancy (97 before stoma closure).

### Factors associated with ‘early’ versus ‘later’ stoma closure

Data were available from NNRD and BAPS-CASS to compare the characteristics of infants with NEC/SIP who underwent stoma closure before or after 9 weeks post formation (comparison based on survey of practice (midpoint of suggested 6 weeks vs 12 weeks, early vs late timings of closure)^7^). Infants who underwent earlier stoma closure were born less preterm and had higher birth weight (table 4). Unsurprisingly, those undergoing earlier stoma closure tended to have a lower weight at the time of operation and slightly higher proportion remained on invasive ventilation. The proportion of those with stoma complications (pre-closure) was higher in the early closure group.

**Table 4:** Comparison of ‘early’ stoma closure (≤9 weeks after formation) and ‘later’ stoma closure (>9 weeks after formation) in infants with NEC/SIP

Characteristics of neonates with NEC/SIP	Early: closure ≤9 weeks after formation		Later: closure >9 weeks after formation	
	NNRD (n=815)	BAPS-CASS (n=42)	NNRD (n=728)	BAPS-CASS (n=39)
Male sex (n, %)	489 (60.0)	24 (57.1)	460 (63.2)	26 (66.7)
Gestation (weeks, median, IQR)	28 (25–31)	26 (25–28)	25 (24–27)	28 (25–31)
Birth weight (g)	986 (761–1486)	925 (685–1294)	764 (650–938)	1000 (780 – 1600)
Age stoma formation (days, median, IQR)	22 (9–43)	20 (10–37)	15 (9–31)	12 (8–27)
Weight stoma formation (g)	1450 (1040–1983)	n/a	916 (745–1200)	n/a
Local stoma* complications	n/a	12 (28.6)	n/a	2 (5.1)
Bowel length duodenojejunal flexure to stoma (cm)†	n/a	47.5 (35–62)	n/a	57.5 (37–85)
Parenteral nutrition 28 days (n, %)	n/a	13 (31.7)	n/a	18 (47.4)
Feed started 28 days (n, %)	n/a	38 (92.7)	n/a	37 (97.4)
Inotropes 2 days pre-closure (n, %)	4.9 (40)	n/a	3.7 (27)	n/a
Parental nutrition 2 days pre-stoma closure (n, %)	498 (61.1)	n/a	399 (54.8)	n/a
Ventilation 2 days pre closure (n, %)		n/a		n/a
None	388 (47.6)		308 (42.3)	
Non-invasive	301 (36.9)		332 (45.6)	
Invasive	122 (15.0)		82 (11.3)	
Missing	4 (0.5)		6 (0.8)	
Steroids in last 7 days (n, %) pre-stoma closure	22 (2.7)	n/a	38 (5.2)	n/a
Age at stoma closure (days, median, IQR)	66 (49–84)	61 (43–81)	108 (92–129)	155 (118–195)
Weight at stoma closure (g)	2330 (1857–2850)	n/a	2767 (2320–3344)	n/a
Duration of stoma	42 (25–53)	n/a	85 (73–105)	n/a

* TPNParenteral nutrition and enteral feeds started missing data for two infants.

† 24 infants from ≤9 weeks and 27 infants from >9 weeks missing data.

BAPS-CASSBritish Association of Paediatric Surgeons Congenital Anomalies Surveillance SystemNECnecrotising enterocolitisNNRDNational Neonatal Research DatabaseSIPspontaneous intestinal perforation

## Discussion

In this study, three national databases were used to evaluate (1) the number and characteristics of neonates undergoing stoma formation for different indications; (2) the timing of stoma closure; (3) the characteristics of infants at the time of closure; and (4) factors associated with timing of closure.

We found that approximately 160–200 babies underwent stoma formation for NEC/SIP in the UK each year: with annual means of 193 and 192 in the NNRD and HES-APC respectively, and 163 in the BAPS-CASS study. For the common year March 2013–March 2014 (online supplemental table 3), the number of babies were 225 (NNRD), 174 (HES-APC) and 163 (BAPS-CASS). The difference between these values is likely to be partly explained by differing geographical coverage (eg, HES-APC being lower than NNRD as it only covers England) and potentially by the BAPS-CASS NEC study having strict inclusion criteria when compared with routinely collected data in the two population-based databases. There will, of course, be inevitable coding errors and missed case reporting. There were fewer neonates with stomas for pathologies other than NEC/SIP, with a mean of 118 neonates/year identified from HES-APC. The much lower value from NNRD (mean of 36 neonates/year) likely reflects the NNRD only holding data on infants in neonatal units and hence not capturing surgery outside of neonatal care, for example, infants discharged home after birth (usually closer to term) and subsequently admitted to specialist children’s surgical units (rather than neonatal units). The true number of neonates with a stoma for conditions other than NEC/SIP is therefore more accurately represented by the HES-APC data.

These data are helpful for planning any future clinical trial of stoma closure timing. They indicate there are approximately 200 neonates with NEC/SIP and 100 neonates with other intestinal malformations who may be eligible for recruitment to a trial each year in the UK. In addition, the sample size calculation for a trial would have to consider missing data at final data endpoint: an important reason for missing data would be infants dying prior to stoma closure. We have shown that there is considerable mortality prior to closure, 197 out of 1543 (13%) and 307 out of 1537 (20%) in the NNRD and HES-APC NEC/SIP cohorts, respectively.

This study demonstrated that stoma closure for infants with NEC/SIP is performed at an average of 2 months post-formation and this was consistent between data sources. We found evidence of variability in practice: among babies with NEC/SIP in the NNRD, BAPS-CASS and HES-APC databases, the upper quartiles were 83, 130 and 124 days, respectively, indicating that many babies wait long periods for closure of their stoma. In addition, a significant number of infants were discharged home prior to stoma closure. This is consistent with the findings from our national survey that demonstrated similar variability in self-reported practice and supports previous calls for a clinical trial to compare early and late stoma closure.^7 10^

The majority of infants in this study appeared to be clinically stable at the time of stoma closure, with only a small proportion continuing to require invasive ventilation, inotropes or had received recent steroids. It, therefore, seems reasonable to conclude that stability for anaesthetic and surgery was a key factor when planning stoma closure, which is again consistent with the findings of our national survey.^7^ These infants did, however, have considerable nutritional demands at the time of closure, with over a half continuing to receive PN, indicating that full enteral feeding could not be achieved with a stoma present. This is supported by previous studies and highlights a potential benefit of earlier closure.^3^ The weight at the time of closure was remarkably similar to the thresholds proposed by surgeons in our national survey (medians of >2500 g (NEC/SIP) and >3000 g (other conditions)). This study does, however, show that some surgeons close stomas earlier without such a weight threshold (lower IQR of 1857g in <6 weeks closure). This further demonstrates variation in practice likely indicative of the underlying uncertainty regarding optimal time for stoma closure.

Infants who underwent earlier stoma closure were born at higher gestation and had higher birth weights (table 4). This likely illustrates the situation whereby extremely preterm infants are deemed too small for early surgery to reverse their stoma, consistent with practice described by clinicians in the national survey.^7^ In addition, clinicians declared that earlier stoma closure would be considered if a stoma was problematic: findings here support this, with a greater proportion of those in the early closure group having local stoma complications (table 4).

These data regarding clinical stability, gestation and weight provide an important insight into some of the complex, inter-related factors that will need to be considered for the design of any clinical trial comparing different stoma closure times. The feasibility of a trial will depend on the population (defined by eligibility criteria) and trial intervention/comparator being acceptable to health professionals and families: data presented, along with other qualitative workstreams in the ToSCiN study, will guide the design of a robust and feasible trial.^7 10^

### Strengths and limitations

While direct comparison of these datasets is complicated by differences in coverage, these data have the key advantage of being population-based, which, to our knowledge, provides the largest and most robust analysis on this topic. The large population allows for a better depiction of clinical practice, regardless of geographical variation and negates the element of bias inherent with small, retrospective studies. Each data source has advantages specific to them, for example, the granularity of BAPS-CASS data, the wide coverage and completeness of the NNRD, and the ability of HES-APC to capture neonates in a variety of healthcare settings and after discharge home.

However, as with all studies using routine data sources, missing data and coding inaccuracies can lead to uncertainty and may mean that this study underestimates the true number of neonates undergoing stoma formation and closure. Our approach of using three, complementary and overlapping data sources minimised this risk.

## Conclusion

Findings from this study indicate a trial to compare early and later stoma closure in neonates is important, owing to variability in current practice around timing of closure and infant weight at time of surgery (thus presenting an opportunity to optimise practice). There appears to be a sufficiently large population of preterm neonates with NEC/SIP undergoing stoma formation to make a UK-based trial feasible. Data presented here around infant characteristics and outcomes will inform the design of a robust trial.

## supplementary material

10.1136/archdischild-2024-327020 online supplemental file 1

## Data Availability

All data relevant to the study are included in the article or uploaded as supplementary information.
