# Supplementary material for: UK neonatal stoma practice: a population study
Source: Arch Dis Child Fetal Neonatal Ed. 2024 Jun 19;110(1):e327020. doi: 10.1136/archdischild-2024-327020 (PMC11671890; doi:10.1136/archdischild-2024-327020)
Supplement: online supplemental file 1 [file fetalneonatal-110-1-s001.pdf]

Supplementary Material

SUPPLEMENTARY METHODS

(i) The National Neonatal Research Database (NNRD)

The NNRD holds data from all infants admitted to National Health Service (NHS) neonatal units in England, Scotland, and Wales (approximately 90,000 infants annually). A formal validation study compared data held in the NNRD with those recorded in Case Record Forms of a multicentre, randomised placebo-controlled trial and found high data completeness (90%) and discordancy rates <5% for the large majority of data points (32/47, 70%) [1].

The NNRD was used to identify infants of all gestational ages born between 01/01/2012 and 31/12/2019 in England and Wales who had a record of a stoma in-situ. This was defined as any baby who had a daily record of a stoma in situ for 2 or more days, with any gastrointestinal diagnosis related to a stoma formation; see Supplementary Table 1 for list of diagnoses and codes. Infants recorded as having an anorectal malformation or Hirschsprung's disease (and hence had a stoma as part of a planned treatment pathway), or who were not cared for entirely in England and Wales, were excluded (Supplementary Figure 1).

We extracted patient characteristics (birthweight, birth year, sex, gestational age at birth); gastrointestinal diagnoses associated with stoma insertion; condition of infant prior to stoma closure (weight on the day prior to the day of stoma closure, receipt of inotropes, parenteral nutrition or respiratory support within 2 days prior to stoma closure, corticosteroids within 7 days prior to stoma closure), whether discharged to surgical centre at any point during care, stoma complications including post-operative intestinal obstruction, post operative wound abscess, small intestinal obstruction due to post-op adhesions and survival to discharge. The timepoint of 2 days prior to closure was chosen, as this was felt to be an accurate reflection of when a clinical team would make the final decision about whether or not the infant was suitable for a planned surgical procedure.

(ii) The British Association of Paediatric Surgeons Congenital Anomalies Surveillance System (BAPS-CASS)

BAPS-CASS has been the UK's principal data collection system with which to study the surgical management of a range of rare congenital anomalies on a population basis. It has conducted several prospective, multi-centre cohort studies since 2006. Two of these were of infants with conditions that frequently require stoma formation, NEC and meconium ileus.

The BAPS-CASS NEC study was conducted between 1/3/2013 and 28/2/2014 in 27 Paediatric surgical centres in the UK and Ireland [2]. The inclusion criteria included any infant with suspected

NEC or SIP where a decision for surgery was made, irrespective of whether they underwent surgery and including any infant subsequently found to have SIP. Infants were excluded if the diagnosis at the time of surgery was not NEC/SIP. Cases were identified by visual inspection of the bowel at surgery, at postmortem, or clinically using the Vermont-Oxford criteria.

The meconium ileus study was conducted between 1/10/2012 and 30/9/2014 in 27 Paediatric surgical centres in the United Kingdom and Ireland [3]. Infants were included if they had bowel obstruction caused by inspissated meconium in the terminal ileum in addition to an established diagnosis of cystic fibrosis.

(iii) Hospital Episode Statistics – Admitted Patient Care (HES-APC)

HES-APC data were obtained from NHS Digital and the records are routinely collected statistical abstracts of inpatient hospital care occurring in NHS hospitals in England, including care occurring outside of neonatal units. Each HES-APC record contains dates of admission and discharge, patient characteristics (which, for birth admissions, include characteristics such as birthweight and gestational age), clinical diagnoses coded using the 10th revision of the International Statistical Classification of Diseases and Related Health Problems (ICD-10), and clinical procedures coded using the OPCS Classification of Interventions and Procedures. The full list of data items collected in HES-APC are described Supplementary table 2. The HES-APC data was used to identify children in England who underwent stoma formation aged <90 days between 01/01/2011 and 31/12/2018. Infants were excluded if they had a diagnosis of an anorectal malformation or Hirschsprung's disease (as the stoma is usually part of a planned treatment pathway).

Statistical analysis

Data were analysed using IBM SPSS 28 and summarised with counts and percentages for categorical variables, or medians (interquartile range [IQR]) for continuous variables. Normality distribution for data was tested using the Shapiro-Wilk test. 'Early' closure was defined as ≤9weeks and 'late' closure was defined as > 9 weeks.

Ethical approval

The study protocol was approved by NHS Research Ethics Committee (REC) IRAS project 278331, REC Reference 20/LO/1227. No patient identifiable information was being used in this study. HES-APC data were obtained from NHS Digital (Data Uses Register reference DARS-NIC-315419-F3W7K).

References

1. Battersby C, Statnikov Y, Santhakumaran S, Gray D, Modi N, Costeloe K, et al. The United Kingdom National Neonatal Research Database: A validation study. PLoS One. 2018;13(8):e0201815.

2. Allin, B., Long, AM., Gupta, A. *et al.* A UK wide cohort study describing management and outcomes for infants with surgical Necrotising Enterocolitis. *Sci Rep* 7, 41149 (2017). https://doi.org/10.1038/srep41149

3. Long A, Jones IH, Knight M, McNally J, Saeed A, Lopes J, et al. Early management of meconium ileus in infants with cystic fibrosis: A prospective population cohort study. Journal of pediatric surgery 2021 Aug 1,;56(8):1287-1292.

SUPPLEMENTARY DATA

Supplementary Table 1: Code numbers relate to as: 1=NEC, 2=volvulus, 3=duodenal pathology, 4=jejunal or ileal pathology, 5=small intestinal obstruction, 6=malrotation or intussusception, 7=intestinal perforation

Clevermed code	Diagnosis	Code
1010683	Necrotising enterocolitis - Suspected	1
10708	Necrotising enterocolitis - perforated	1
15809	Necrotising Enterocolitis - confirmed	1
15809	Necrotising enterocolitis	1
15808	Intestinal obstruction : volvulus	2
16212	Congenital absence, atresia and stenosis of duodenum	3
16213	Duodenal atresia	3
16213	Duodenal atresia / stenosis	3
16214	Jejunal atresia / stenosis	4
16215	Congenital absence, atresia and stenosis of ileum	4
16216	Ileal atresia / stenosis	4
16217	Congenital absence, atresia and stenosis of small intestine	5
10977	Small intestinal obstruction due to stricture (acquired)	5
10973	Small intestinal atresia / stenosis (specify)	5
10975	Small intestinal obstruction due to internal hernia	5
10976	Small intestinal obstruction due to post-op adhesions	5
10977	Small intestinal obstruction due to stricture (acquired)	5
10978	Small intestinal obstruction due to volvulus	5
11147	Volvulus	2
15808	Intestinal obstruction : volvulus	2
16231	Malrotation of the intestine	6
15798	Intussusception / obstruction	6
15799	Intestinal obstruction ? cause	5
15817	Neonatal intestinal perforation	7
15819	Neonatal ileum perforation	7
15821	Neonatal terminal ileum perforation	7
11010244	Intestinal perforation - not NEC	7
11217	Closure of jejunal perforation	7
11222	Closure of small intestine/ileal perforation	7

Suppmentary Table 2: Case Definitions for HES analysis

Anorectal Malformations and Hirschsprungs Disease (at any diagnostic level i.e. not just primary diagnosis and present at any admission)

Collective number to be recorded in Table 1 and Supplementary 1/2

- Q431 Hirschsprung's disease OR
- Q423 Cong absence atresia and stenosis anus without fistula OR
- Q422 Cong absence atresia and stenosis anus with fistula OR
- Q437 Persistent cloaca

Defining NEC and intestinal perforation group
(at any diagnostic level during episode of stoma formation)

- P77 Necrotising enterocolitis OR
- P780 Perinatal intestinal perforation OR
- K631 Perforation of intestine (nontraumatic)

But NOT including one of these diagnostic codes during the same episode

- Q793 Gastroschisis OR
- Q439 Congenital malformation of intestine, unspecified OR
- P760 Meconium plug syndrome OR
- Q438 Other specified congenital malformations of intestine OR
- Q411 Congenital absence, atresia and stenosis of jejunum OR
- Q412 Congenital absence, atresia and stenosis of ileum OR
- E841 Cystic fibrosis with intestinal manifestations OR
- P75 Meconium ileus in cystic fibrosis OR
- Q433 Congenital malformations of intestinal fixation OR

Defining other intestinal malformation group

- All other infants with stoma not included in the above two groups

Supplementary Figure 1

Supplementary Figure 1a: Flowchart depicting the exclusion and data cleansing process. Neonates with a record of a stoma in situ in England and Wales, within the National Neonatal Research Database, 1st January 2012 to 31st December 2019

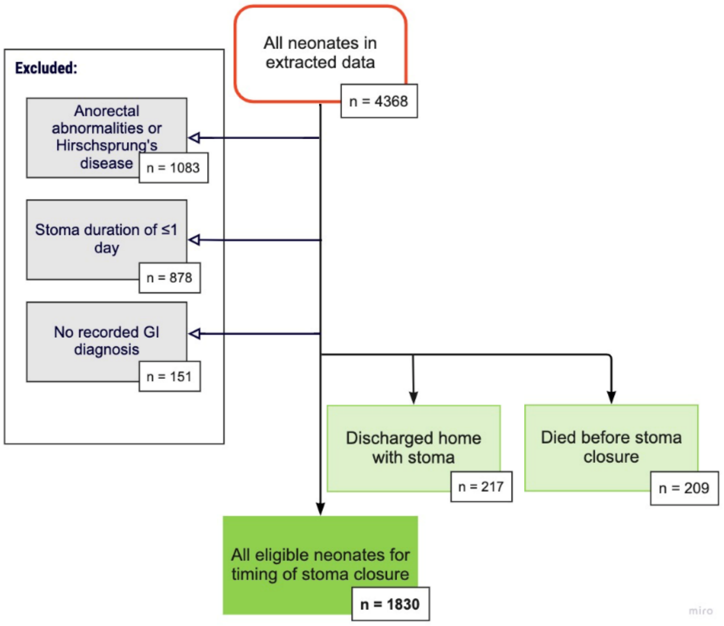

Supplementary Figure 1b: Flowchart of infants included in the BAPS-CASS NEC/SIP study

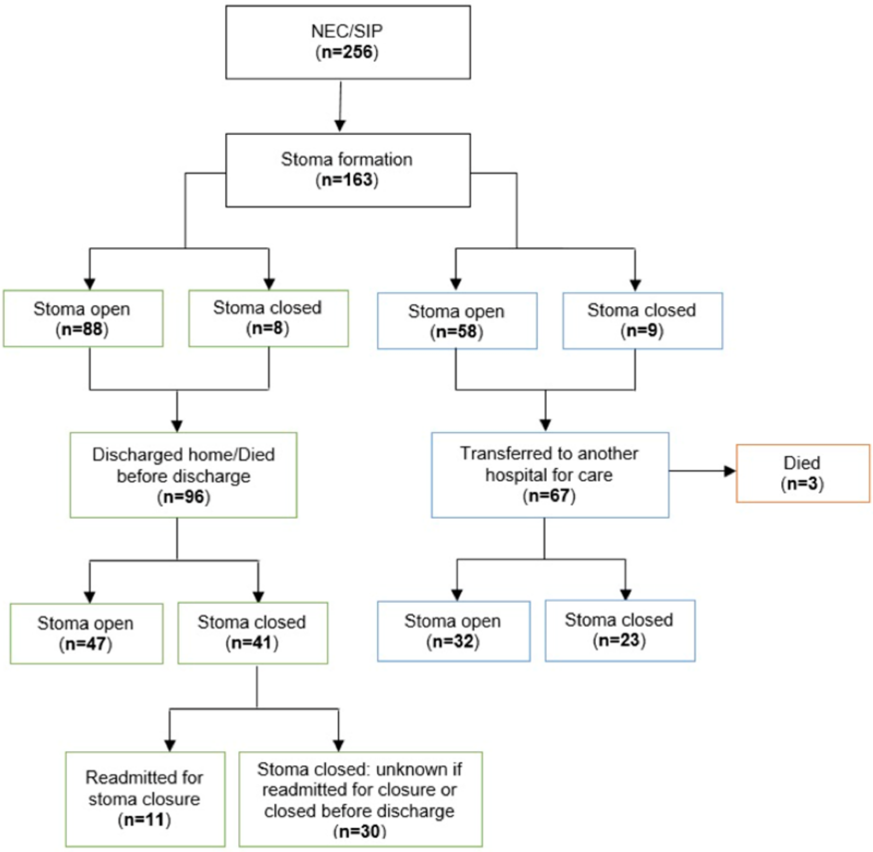

Supplementary Figure 1c: Flowchart of infants included in HES-APC analysis

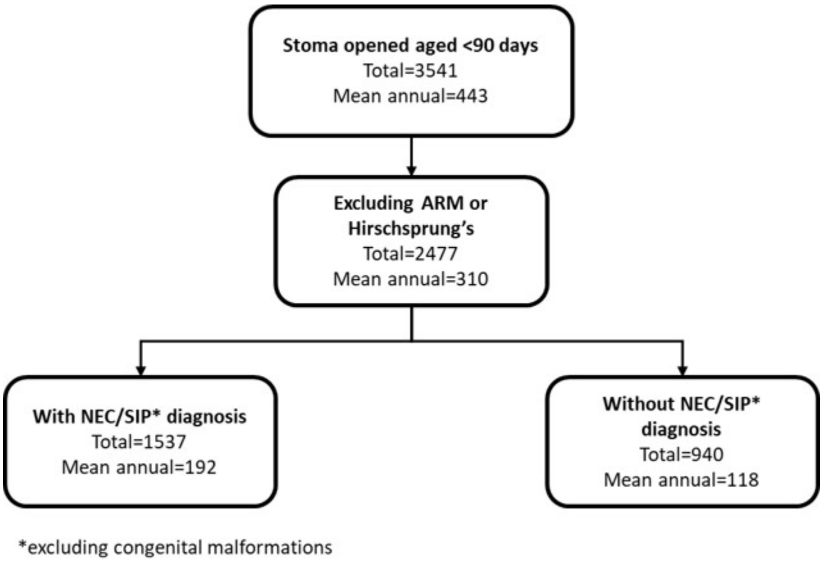

Supplementary Table 3: Indications for stoma formation for common year (March 2013 - March 2014) and summary of data source characteristics

Data Source		NNRD	HES	BAPS-CASS
Coverage		England/Wales	England	UK
Setting		All neonatal units	All NHS settings	Centres providing neonatal surgery
Total infants	n=	62519		256
Total infants with stoma	n=	290	423	163
Anorectal Malformations & Hirschsprungs (planned treatment pathways: excluded)	n=	32	131	n/a
NEC/Intestinal Perforations	n=	225	174	163
Other intestinal malformations	n=	33	118	n/a

Supplementary Figure 2: Histogram of stoma closure time for meconium ileus (BAPS-CASS)

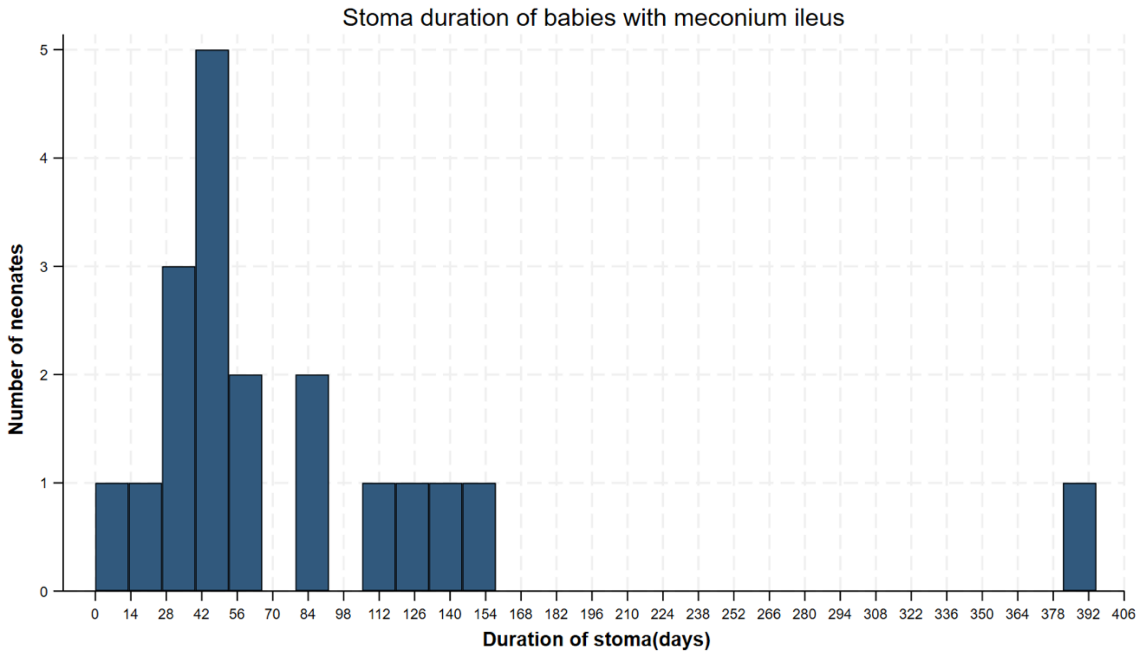

The median (IQR) time from stoma formation to closure was 51 days (36 – 106).

Supplementary table 4: Characteristics of infants from the National Neonatal Research Database (NNRD) who were discharged from neonatal care or died prior to stoma closure

		**Necrotising enterocolitis**	**Other malformations**
**Discharged prior to stoma closure**	Number of neonates (n)	N=178	N=39
	Duration of stoma prior to discharge (median, IQR)	49 [33-80]	28 [17-49]
	Gestation age at birth (median, IQR)	29 [26-33]	36 [30-38]
	Birth weight in g (median, IQR)	1110 [823-1790]	2160 [1135-3090]
**Death prior to stoma closure**	Number of neonates (n)	197	12
	Duration of stoma prior to death (median, IQR)	16 [7-40]	16 [4-39]
	Gestation age at birth (median, IQR)	25 [24-27]	26 [24-34]
	Birth weight in g (median, IQR)	717 [610-870]	720 [585 – 1935]
